# Genome Sequence of *Southern tomato virus* in Asymptomatic Tomato ‘Sweet Hearts’

**DOI:** 10.1128/genomeA.01374-16

**Published:** 2017-02-16

**Authors:** Ricardo I. Alcalá-Briseño, Sevgi Coşkan, Maria A. Londoño, Jane E. Polston

**Affiliations:** Department of Plant Pathology, University of Florida, Gainesville, Florida, USA

## Abstract

The genome sequence of *Southern tomato virus* in asymptomatic *Solanum lycopersicum* ‘Sweet Hearts’ (STV-Florida) in Florida was assembled from small RNAs sequenced by Illumina RNA-seq. The STV-Florida genome shared 99.0 to 99.9% similarity with full genome sequences from Bangladesh, China, Mexico, and the United States (Mississippi and North Carolina).

## GENOME ANNOUNCEMENT

*Southern tomato virus* (STV), a virus species in the genus *Amalgavirus*, in the family *Amalgaviridae*, has one known host, tomato [*Solanum lycopersicum* (L.)] (1). STV has a 3,437-bp double-stranded RNA genome with two overlapping coding regions. The smaller region codes for a putative 42-kDa (p42) coat protein (CP), and the larger uses a +1 ribosomal frameshift to encode a 121-kDa (p121) RNA-dependent RNA polymerase (1). No virion has been associated with STV (1). STV is only known to be transmitted vertically, with rates of transmission through seed reported as high as 90% (1). STV was reported for the first time in Mexico (Colima) and the United States (California and Mississippi) in 2005, and the first genome sequence was published in 2009 (1). Since then it has been reported from multiple locations around the world (2–6). In 2013, STV was detected in asymptomatic plants of *S. lycopersicum* ‘Sweet Hearts’ in Florida, using small RNA sequencing and single reads in Illumina HiSeq 2000 (Macrogen, Seoul, South Korea). 

The RNA-seq was assembled using Velvet version 1.2.09 (7) and analyzed by BLAST to identify homology to viral sequences in the nonredundant NCBI database (8). BLAST results showed homology to STV (9). STV-Florida from ‘Sweet Hearts’ was reconstructed with Bowtie and Geneious version R9.1.3 using 8,682 reads and 127 contigs aligned to a reference sequence (NC_011591) to produce a full genome sequence (3,438 nucleotides [nt]) (KX949574). The STV-Florida genome has a 138-nt 5′ untranslated region (UTR) and a 110-nt 3′ UTR, with two coding regions: one that codes for a putative 377–amino acid (aa) protein that is identical to the CP (p42) of STV from Mexico (YP_002321510) and a second that codes for a putative 1,062-aa protein produced by a ribosomal frameshift, which is identical to p121 of STV from Mexico (YP_002321510). Pairwise comparison (Muscle and Geneious version R9.1.3) of full genome of STV-Florida from ‘Sweet Hearts’ with those of five isolates from Bangladesh, China, Mexico, and United States (Mississippi and North Carolina) (KT634055, KT438549, EF442780, EU413670, and KT852573) showed similarities of 99.0 to 99.9%. While diversity was low, these sequences could have been obtained from closely related cultivars that had exchanged STV isolates recently through genetic crossing. Amplicons of 440 nt (positions 993 to 1432) were generated using STV-F/STV-R primers (1) from two hybrid cultivars, ‘Sweet Hearts’ (STV-SH-17m) and ‘Agriset 761’ (STV-Agr761-11-7), as well as two open-pollinated cultivars, ‘Mexico Midget’ (STV-MM-2-6) and ‘Roma’ (STV-R-11-6). Being open-pollinated, ‘Mexico Midget’ and ‘Roma’ are the least likely to have exchanged genetic material in the last few decades. Pairwise comparison of these partial sequences, plus the equivalent sequence of the five full-genome STV sequences, revealed a total of only six nucleotide changes: one in STV-SH-17m, three in STV-MM-2-6, and two in STV_CN12 (unnamed cultivar from China) (KT438549), resulting in an overall similarity of 99.7%.

### Accession number(s).

The assembled genome of STV-Florida and amplicon sequences were deposited in GenBank under the following accession numbers: KX949574 (STV-Florida), KX949570 (STV-Agr761-11-17), KX949571 (STV-MM-2-6), KX949572 (STV-R-11-6), and KX949573 (STV-SH-17m).
